# Familial deletion 18p syndrome: case report

**DOI:** 10.1186/1471-2350-7-60

**Published:** 2006-07-14

**Authors:** Bruno Maranda, Nicole Lemieux, Emmanuelle Lemyre

**Affiliations:** 1 Service de génétique médicale, Département de Pédiatrie, CHU Ste-Justine, Université de Montréal, Montréal, Canada; 2 Département de Pathologie, CHU Ste-Justine, Université de Montréal, Université de Montréal, Montréal, Canada

## Abstract

**Background:**

Deletion 18p is a frequent deletion syndrome characterized by dysmorphic features, growth deficiencies, and mental retardation with a poorer verbal performance. Until now, five families have been described with limited clinical description. We report transmission of deletion 18p from a mother to her two daughters and review the previous cases.

**Case presentation:**

The proband is 12 years old and has short stature, dysmorphic features and moderate mental retardation. Her sister is 9 years old and also has short stature and similar dysmorphic features. Her cognitive performance is within the borderline to mild mental retardation range. The mother also presents short stature. Psychological evaluation showed moderate mental retardation. Chromosome analysis from the sisters and their mother revealed the same chromosomal deletion: 46, XX, del(18)(p11.2). Previous familial cases were consistent regarding the transmission of mental retardation. Our family differs in this regard with variable cognitive impairment and does not display poorer verbal than non-verbal abilities. An exclusive maternal transmission is observed throughout those families. Women with del(18p) are fertile and seem to have a normal miscarriage rate.

**Conclusion:**

Genetic counseling for these patients should take into account a greater range of cognitive outcome than previously reported.

## Background

Deletion of the short arm of chromosome 18, del(18p), is now a well established chromosomal aberration. It has been first described by the French geneticist Jean de Grouchy in 1963 [1]. Since then, more than one hundred have been reported [2].

Phenotypic manifestations of this deletion are very sparse at birth. The female to male ratio is 3:2 and birth weight averages 2600 g. The most frequent abnormalities consist of mild to moderate growth deficiency, mental retardation, microcephaly, ptosis, epicanthal folds, low nasal bridge, hypertelorism and large protruding ears. Holoprosencephaly and clinodactyly of the fifth finger is observed in about 10% and 20% of the cases respectively. Recent evidences have suggested an association with growth hormone deficiency that responded well to hormone supplementation [3]. Mental retardation is mild to severe with an average intellectual quotient (IQ) between 45 and 50. There is a significant discrepancy between verbal and non-verbal performance, verbal performance being more severely affected [2,4,5]. Dystonias are also reported [6].

Most cases of deletion 18p are supposed to originate from *de novo *deletions, which accounts for 85% of cases [7]. The remainder is suspected to come from unbalanced familial transmission of structural rearrangements. New cytogenetic techniques have shown one case of an unbalanced subtelomeric translocation causing del(18p) [8]. Until now, there have been 5 reported families with transmission of del(18p) from a mother to a child (summarized in table 1). Here we report a familial transmission of del(18p) from a mother to her two daughters with variable intellectual outcome and better verbal performance which differs from the classical del(18p) phenotype.

**Table 1:** 18p deletion: familial transmission cases.

Author	Family member	Chromosomal anomaly	Mental retardation	Short Stature	Dysmorphic features	Notes
Uchida[9]	Mother	del(18p)	+/-	+	+	88% mosaicism
	Son	del(18p)	+	+	+	DQ = 65–70
	Daughter	del(18p)	?	?	?	Stillborn, cebocephalia
Velagaleti[10]	Mother	del(18p11.2)	+	+	-	
	Daughter	del(18p11.2)	+	+	+	IQ = 69
Tonk[20]	Mother	del(18p11.2)	+	-	+	Mild MR
	Fetus	del(18p11.2)	?	?	?	Cyclopia, holoprosencephaly
Tsukahara[11]	Mother	del(18p11.2)	+/-	-	+	
	Daughter	del(18p11.23)	+/-	-	+	DQ = 74
	Son	del(18p11.2)	+/-	-	+	11 months
Rigola[18]	Mother	del(18p11.3)	+	-	+	
	Fetus	del(18p), t(X, Y)	?	?	?	No anomalies at autopsy
This report	Mother	del(18p11.2)	+	-	+	IQ = 43
	Proband	del(18p11.2)	+	+	+	Moderate MR
	Daughter	del(18p11.2)	+/-	-	+	Borderline to mild MR

DQ, developmental quotient; IQ, intelligence quotient; MR, mental retardation.

## Case presentation

### Proband

The proband is a girl now aged 12 years. Pregnancy was noticeable only on the basis of a placenta previa without complications. The mother was 22. Birth weight was 2950 g. The child was brought to medical attention at the age of 4 because of global developmental delay. Past medical history is positive for recurrent otitis media that caused moderate conductive hearing loss, which required tympanostomy. She started a stimulation program at the age of 4 because of developmental delay. Cognitive performance is in agreement with moderate mental retardation (IQ between 40 to 55) according to the Weschler intelligence scale for children, third edition (WISC-III). There is no difference between verbal and non-verbal functions.

**Figure 1:**
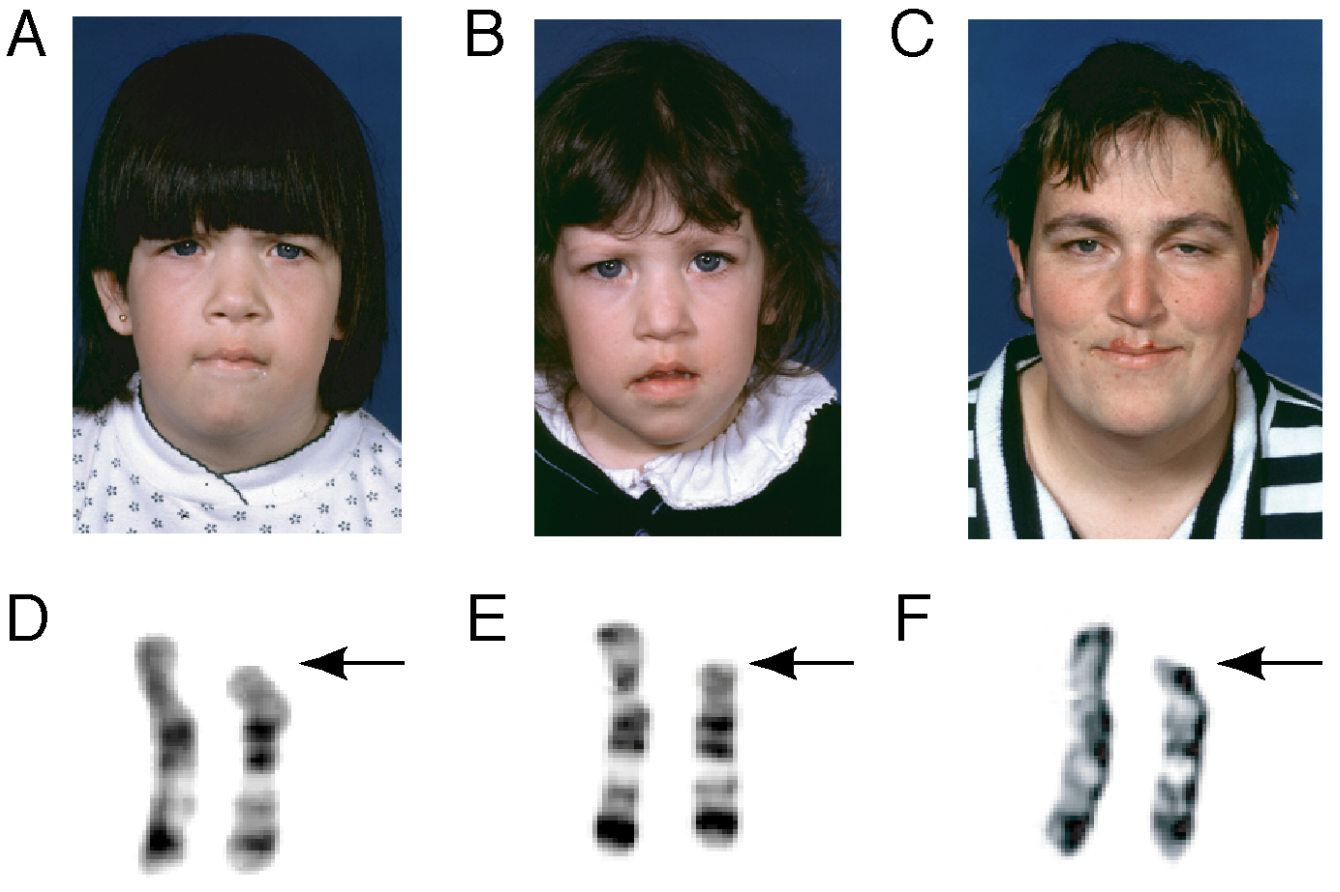
Phenotypical and chromosomal characteristics of the affected members of the family showing 18p deletion (arrow). Proband (A, D), sister (B, E) and mother (C, F).

Phenotypic manifestations are the following: weight is at the 50^th ^centile, height at 3rd centile and head circumference at 50^th ^centile. The face is rectangular, hair implantation is low, the ears are large and protruding, the mouth is large with a prominent philtrum and a high-arched palate (Figure 1A). Small hands and clinodactyly of the fifth fingers were noted. Neurological examination is normal.

Cerebral imaging revealed asymmetrical lateral ventricles without any associated lesion. Renal ultrasound was performed and revealed no abnormalities. Endocrine evaluation was also normal, including thyroid stimulating hormone and insulin-like growth factor 1. Immune function was adequate with immunoglobulin A in normal range.

### Second daughter

The second daughter is now aged 9. Pregnancy was uneventful and birth weight was 3630 g. Early development was normal and she walked at 1 year of age. Psychological evaluation with a WISC-III test indicated that she has a non-verbal IQ in the mild mental retardation range (55 to 70) and a verbal IQ in the borderline range (70 to 85). However, this difference is not significant. Her global IQ is in the borderline range but close to mild mental retardation.

On examination, her weight is at the 25^th ^percentile, height is at the 10^th ^percentile and head circumference is at the 75^th ^percentile. Phenotypical observation revealed the following features: low posterior hairline, large protruding ears, rectangular face, epicanthal folds, large mouth with prominent philtrum, high-arched palate and downturning corners of the mouth (Figure 1B). Small hands and clinodactyly of the fifth fingers were also present.

Like her sister, renal ultrasound, endocrine function and immune function were all normal.

### Mother

The mother's history is noticeable for learning difficulties especially in mathematics. Her global IQ is 43 consistent with moderate mental retardation. There is a significant difference (p = 0,05) between verbal and non-verbal IQ, 52 and 42 respectively. She leads an active and independent life. Her pubertal development is described as normal with menarche at age 13. She had a miscarriage at 16 weeks of pregnancy before her two daughters. The mother does not complain of dystonia.

Her height is 157 cm, which is around the fifth percentile. She has a rectangular face, low posterior hairline, downturning corners of mouth with prominent philtrum and a high-arched palate (Figure 1C). Unilateral ptosis is present. She showed clinodactyly of her fifth fingers as well. Immune and endocrine functions were normal.

### Cytogenetics

**Figure 2:**
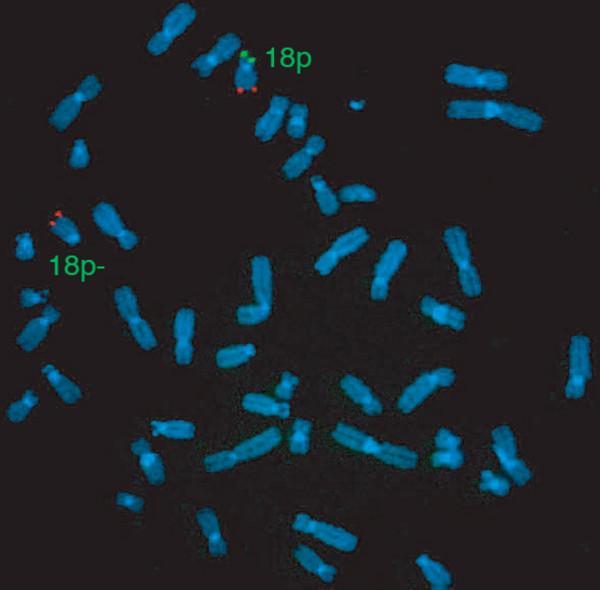
In situ hybridization. 18p (green) and 18q (red) with subtelomeric probes showing 18p deletion in the proband.

Chromosome analysis from peripheral blood lymphocytes was carried out using GTG banding procedure on the whole family. The proband analysis revealed a 46, XX, del(18)(p11.2) complement in all 14 cells analyzed (Figure 1D). Fragile X syndrome was ruled out by polymerase chain reaction amplification. Proband's sister and mother revealed the same deletion (Figure 1E and 1F). Father's chromosomal analysis showed a 46, XY karyotype. In the proband, further investigation using fluorescence *in situ *hybridization (FISH) with 18p/18q subtelomeric probes on 20 mitosis was in agreement with cytogenetic results and showed a terminal deletion of 18p (Figure 2) with no hybridization of 18p subtelomeric probe on any other chromosome. Maternal grandfather's karyotype was normal but the maternal grandmother was unavailable.

### Discussion

This paper presents the sixth family with transmission of an 18p deletion. Phenotypical features were quite similar throughout the family and in accordance with the usual phenotype of del(18p). Short stature, low hairline, rectangular face, high-arched palate, prominent philtrum and clinodactyly of the fifth fingers were present throughout the family. The girls shared also large protruding ears, large mouth and small hands.

Another feature consistent throughout the familial del(18p) cases is poor intellectual outcome. Uchida et al. [9], described a 88% mosaic mother for del(18p) with an IQ of 75. Her child also had a developmental delay, performing at 65–70% of normal. Velagaleti et al. [10], presented a mother having an 18p11.2 deletion with mild mental retardation. Her daughter's psychosocial evaluation revealed a verbal IQ of 63 and a full scale IQ of 69 consistent with mild mental retardation. Tsukahara et al. [11], showed a Japanese woman also bearing del(18p11.2) who finished her junior high school. Her son presents a slight developmental delay at 11 months and her daughter has a DQ of 74 with significant speech delay placing them on the borderline intellectual functioning level.

We would like to underscore the fact that among the families presented above, the intellectual outcome is relatively constant throughout the same family. The family we report here is in discrepancy with this statement. The mother and proband share moderate mental retardation while the youngest daughter has borderline to mild mental retardation. This represents a significant difference among members of the same family afflicted by an identical chromosomal deletion. Such variability has been observed in other cases of familial deletions [12-14]. Even greater variability among family members with del(18p) is exemplified by the two holoprosencephaly cases. Mental retardation would have been expected in such cases of extreme brain dysmorphogenesis.

Del(18p) cases were associated with poorer verbal performance than non-verbal [2,4,5]. Our family equally differs in this regard. The mother has better verbal abilities and the two daughters do not display differences between verbal and non-verbal abilities. Therefore, none of our family members with del(18p) present the previously described verbal difficulties.

The six families with del(18p) were maternally transmitted. No paternal transmission has been described to our knowledge although molecular characterization of 18p *de novo *deletions showed an equal distribution of maternal and paternal distributions [15]. However, it has been observed that women transmit unbalanced chromosomes aberrations more frequently than men [16]. Since no imprinting center is known on chromosome 18 [17], a deleterious paternal allele with del(18p) would be a surprising phenomenon. Whether this observation is simply a statistical bias because of the small numbers involved or has a real cytogenetic background that could affect male fertility is still unknown but demands consideration.

Rigola et al. [18], discussed the fact that none of the cases published had a history of spontaneous abortions. This report presents the first mother with del(18p) syndrome having a miscarriage. This event happened at the 16^th ^week of her first pregnancy and no analysis was performed on the fetus. Among the 9 clinical pregnancies of these 6 women, one aborted spontaneously which is in agreement with the 10 to 15% rate of miscarriage in the general population. Fertility of women with del(18p) syndrome seems to be normal although gonadal failure has been previously reported among those patients [19].

## Conclusion

This report sheds new lights on the familial del(18p) syndrome. Cognitive performance may be more variable than previously suggested within the same family. Verbal abilities could be superior to non-verbal abilities in some individuals. These women are fertile with a miscarriage risk equivalent to the general population. Among the six del(18p) families described, exclusive maternal transmission was seen. Genetic counseling for these patients should take into account these new data, especially in regard of a wider variability of intellectual outcomes and better verbal performance.

## Competing interests

The author(s) declare that they have no competing interests.

## Authors' contributions

BM designed the study, collected data and drafted the manuscript.

NL performed cytogenetic analysis and revised the manuscript.

EL collected data, performed cytogenetic analysis and revised the manuscript.

## Pre-publication history

The pre-publication history for this paper can be accessed here:


